# Polynucleotide phosphorylase is implicated in homologous recombination and DNA repair in *Escherichia coli*

**DOI:** 10.1186/s12866-017-0980-z

**Published:** 2017-04-04

**Authors:** Thomas Carzaniga, Giulia Sbarufatti, Federica Briani, Gianni Dehò

**Affiliations:** 1 Dipartimento di Bioscienze, Università degli Studi di Milano, via Celoria 26, Milan, 20133 Italy; 2 Present address: Dipartimento di Biotecnologie mediche e medicina traslazionale, Università degli Studi di Milano, via F.lli Cervi 93, Segrate, MI 20090 Italy; 3 Present address: Eurofins BioPharma Product Testing Italy, Eurofins Biolab srl, via Bruno Buozzi, 2, Vimodrone, 20090 Italy

**Keywords:** Polynucleotide phosphorylase, Genetic recombination, DNA repair, Mutagenesis, RNA metabolism

## Abstract

**Background:**

Polynucleotide phosphorylase (PNPase, encoded by *pnp*) is generally thought of as an enzyme dedicated to RNA metabolism. The pleiotropic effects of PNPase deficiency is imputed to altered processing and turnover of mRNAs and small RNAs, which in turn leads to aberrant gene expression. However, it has long since been known that this enzyme may also catalyze template-independent polymerization of dNDPs into ssDNA and the reverse phosphorolytic reaction. Recently, PNPase has been implicated in DNA recombination, repair, mutagenesis and resistance to genotoxic agents in diverse bacterial species, raising the possibility that PNPase may directly, rather than through control of gene expression, participate in these processes.

**Results:**

In this work we present evidence that in *Escherichia coli* PNPase enhances both homologous recombination upon P1 transduction and error prone DNA repair of double strand breaks induced by zeocin, a radiomimetic agent. Homologous recombination does not require PNPase phosphorolytic activity and is modulated by its RNA binding domains whereas error prone DNA repair of zeocin-induced DNA damage is dependent on PNPase catalytic activity and cannot be suppressed by overexpression of RNase II, the other major enzyme (encoded by *rnb*) implicated in exonucleolytic RNA degradation. Moreover, *E. coli pnp* mutants are more sensitive than the wild type to zeocin. This phenotype depends on PNPase phosphorolytic activity and is suppressed by *rnb*, thus suggesting that zeocin detoxification may largely depend on RNA turnover.

**Conclusions:**

Our data suggest that PNPase may participate both directly and indirectly through regulation of gene expression to several aspects of DNA metabolism such as recombination, DNA repair and resistance to genotoxic agents.

## Background

Polynucleotide phosphorylase (PNPase, polyribonucleotide nucleotidyltransferase, EC 2.7.7.8), an enzyme widely conserved in Bacteria and in eukaryotic organelles of bacterial origin, reversibly catalyses the 3′-to-5′ phosphorolysis of polyribonucleotides, releasing nucleoside diphosphates (NDPs) and the reverse template-independent 5′-to-3′ polymerization of nucleoside diphosphates, releasing inorganic phosphate (Pi) [1, 2]. The original interest for the RNA polymerizing activity of this enzyme [3] was superseded by the J. Hurwitz discovery of DNA-dependent RNA polymerase (reviewed by [4]) and RNA degradation was since thought of as the main in vivo activity of PNPase [5]. Its RNA polymerizing activity has also been implicated in PNPase-dependent RNA decay, as in *Escherichia coli* polyadenylation and heteropolymeric tailing of RNA 3′-ends performed by polyadenylpolymerase (PAP) and PNPase, respectively, target bacterial RNAs to degradation [6–8]. A wealth of evidence accumulated in the last decades indicates that the key role of PNPase in vivo is to modulate the abundance of a number of mRNAs and small RNAs (sRNAs), and thus expression of many genes (reviewed by [2]).

Remarkably, PNPase can catalyse both DNA phosphorolysis and template independent synthesis of DNA from dNDPs [9–15]. The latter enzymatic property was exploited in the early era of molecular biology for the synthesis of oligodeoxyribonucleotides but was not generally considered to play a role in vivo. However, features that link this enzyme to DNA metabolism have emerged. For example, PNPase deficient *E. coli* mutants are more sensitive to UV [16] and exhibit a lower mutation frequency [17]. Such phenotypes have been thought of as a direct or indirect result of PNPase RNA-degrading activity; however, the recent observation that in *Bacillus subtilis* not only PNPase is implicated in DNA repair but also is part of the RecN repair complex [13, 14, 16, 17], raises the possibility that its DNA degradative and/or polymerizing activities may be implicated in DNA recombination, repair and mutagenesis.

Homologous recombination (HR) was first identified as a mechanism that assorts genes on homologous chromosomes at meiosis, thus contributing to the generation of genetic variability at the population level. Genetic studies on fungi, which led to the pioneering Holliday’s model, implicated HR process in mismatch repair, whereas studies on recombination and DNA repair deficient mutants in *Escherichia coli* readily highlighted that the two phenomena are inextricably intertwined and that multiple pathways and mechanisms are implicated in these processes [18]. Finally, as DNA damage, in particular double strand breaks (DSB) generated during progression of the replicative fork, represent an obstacle to completion of chromosome replication, it was recognised that recombination and DSB repair (DSBR) are essential for cell viability. It is now widely accepted that HR is a housekeeping process implicated in the maintenance of genome integrity both during and after DNA replication [18].

Given the pivotal role of recombination and DNA repair for cell survival and homeostasis, it is not surprising that such processes cross talk with other central cellular pathways. In this work we show that PNPase, an enzyme typically implicated in RNA turnover, may play a role both in transduction-mediated HR and in error prone DNA repair, thus providing further evidence for the connections between RNA and DNA metabolism.

## Results

### Transduction frequency is reduced in *E. coli* Δ*pnp* mutant

PNPase has recently been implicated in recombination and DNA repair in *Bacillus subtilis* [14]. We tested the potential involvement of PNPase in homologous recombination in *E. coli* by assessing the frequency of P1-mediated generalized transduction in the presence and absence of PNPase. Isogenic wild type and Δ*pnp* strains auxotrophic for tryptophan (Δ*trpE*::*kan*) or leucine (Δl*euA*::*kan*) were infected with a lysate of P1 HFT phage grown in the prototrophic strain C-1a and prototrophic transductants were selected on M9 glucose agar minimal medium, as described in Materials and Methods. All prototrophic transductants tested turned out to be Kan^S^, thus indicating that recombination had occurred at the homologous loci in both wild type and Δ*pnp* srains. The results of these experiments reported in Table 1 show that for both independent auxotrophy markers transduction frequency was 5–6 folds lower in the *pnp* mutant.

**Table 1:** P1 transduction in Δ*pnp* strains

Transduced marker	*trpE*				*leuA*			
Chromosomal *pnp* allele	Adsorbed phage^a^	m.o.i.^b^	TF^c^ (×10^7^)	TF fraction^d^	Adsorbed phage^a^	m.o.i.^b^	TF^c^ (×10^7^)	TF fraction^d^
wild type	0.98 (±0.01)	5.87×10^-2^	37.0 (±1.4)	1.00	0.98 (±0.01)	5.66×10^-2^	23.0 (±1.2)	1.00
Δ*pnp-751*	0.97 (±0.01)	5.84×10^-2^	6.9 (±0.28)	0.19	0.97 (±0.01)	5.81×10^-2^	3.7 (±0.17)	0.16

^a^adsorbed phage is input phage - unadsorbed phage as assayed 20 min post infection

^b^multiplicity of infection (m.o.i.) is the ratio of phage to bacterial cells

^c^transduction frequency (TF) is the ratio of transductants to adsorbed phage. Average and standard deviation of three experiments are reported

^d^ratio to TF of wild type strain

Recombination is only one of the steps required for transduction and it could be argued that PNPase may affect efficiency of upstream events such as phage adsorption and/or DNA injection. However, P1 adsorption was comparable in wild type and *pnp* ^−^ strains (Table 1) whereas P1 efficiency of plating (e.o.p.) was slightly lower (0.79), with a smaller plaque size, in the two Δ*pnp* recipients than in their isogenic *pnp* ^*+*^ strains. Even assuming that the lower e.o.p. exclusively depends on less efficient DNA injection rather than on downstream steps in phage growth cycle (as suggested by the smaller plaque size), it does not account for the 5–6 fold reduction in transduction efficiency. It seems thus possible that PNPase contributes to some extent to the efficiency of recombination of transduced DNA.

PNPase is composed of a catalytic core and a C-terminal region composed of two RNA binding domains (RBDs) KH and S1 [19–21]. To test whether either or both catalytic and RNA binding activities were implicated in transduction efficiency, we ectopically complemented the above Δ*pnp* strains with plasmids expressing the wild type PNPase, PNPase mutants lacking either or both the KH and S1 domains [22, 23], and a PNPase with a point mutation in the catalytic sites (PNPase^S438A^) known to abolish phosphorolytic activity [24, 25]. As shown in Table 2, transduction efficiency in the non-complemented Δ*pnp* strain (harbouring the empty vector) and in strains expressing PNPase lacking either RBD was from about 3–5 fold lower than in the *pnp* ^+^-complemented strain, whereas the catalytically inactive mutation *pnp* ^S438A^ marginally (if at all) affected recombination (1.2–1.7 fold decrease). Surprisingly, the lack of both KH and S1 domains strongly reduced (25–45 fold) the recovery of prototrophic transductants. It thus appears that nucleic-acid binding activity is required to regulate transduction efficiency and that the presence of an enzyme devoid of both RBDs impairs recombination more than the mere lack of PNPase.

**Table 2:** Efficiency of P1 transduction: complementation by different *pnp* mutant alleles

Plasmid^a^	*pnp* allele on plasmid	*trpE*		*leuA*	
		TF^b^ (×10^7^)	TF fraction^c^	TF^b^ (×10^7^)	TF fraction^c^
pAZ101	wt	2.53 (±0.09)	1.00	4.40 (±0.35)	1.00
pGZ119HE	none	0.75 (±0.20)	0.30	1.34 (±0.30)	0.30
pAZ1112	S438A	2.07 (±0.52)	0.82	2.70 (±0.46)	0.60
pAZ1113	ΔKH	0.55 (±0.15)	0.22	1.18 (±0.24)	0.26
pAZ1114	ΔS1	0.53 (±0.19)	0.21	1.19 (±0.25)	0.27
pAZ133	ΔKH-S1	0.06 (±0.02)	0.02	0.17 (±0.06)	0.04

^a^in C-5691 (Δ*pnp*) strain

^b^transduction frequency (TF) is the ratio of transductants to adsorbed phage

^c^ratio to TF of wild type strain

### *E. coli* Δ*pnp* is more sensitive to the genotoxic agent zeocin

The substrates of transduction-mediated recombination are the circular chromosome and a homologous linear dsDNA fragment [26], likely implicating the RecBCD-dependent recombination pathway [27], which is promoted by free dsDNA ends [28]. We thus addressed whether PNPase also participates in repair processes of DSBs induced by zeocin (phleomycin D1), a glycopeptide of the bleomycin/phleomycin antibiotic family, known to cause DSBs in vivo [29].

**Fig. 1:**
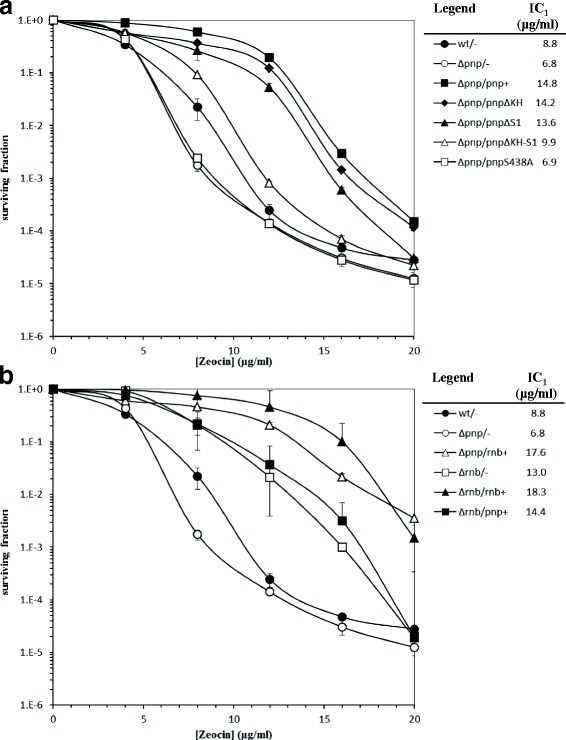
Survival of *E. coli pnp* and *rnb* mutants to chronic zeocin treatment. Bacterial cultures grown overnight at 37 °C in LD broth with chloramphenicol were serially diluted and plated onto LD-agar with chloramphenicol and 0, 4, 8, 12, 16, 20 μg/ml zeocin; the plates were incubated at 37 °C for 2 days. The surviving fraction was calculated as the ratio of colony forming units (CFUs) at different zeocin concentration to the untreated control. The means of at least two independent experiments and the standard deviations (*error bars*) are shown. Zeocin inhibitory concentration giving 1% survival (IC_1_) was extrapolated from the survival curves and reported as μg/ml on the *right column* in the panel legends. **a** Complementation of *E. coli pnp* deletion mutant with different *pnp* alleles. **b** Complementation of *E. coli pnp* and *rnb* deletion mutants. The *pnp* or *rnb* alleles harbored by the host chromosome and or by the plasmids are indicated by the *labels* before and after the *slash*, respectively. Bacterial strains: wt, C-1a; Δpnp, C-5691; Δrnb, C-5981. Plasmids: −, pGZ119HE; pnp+, pAZ101; rnb+, pAZ1115; pnpΔKH, pAZ1113; pnpΔS1, pAZ1114; pnpΔKH-S1, pAZ133; pnpS438A, pAZ1112

Firstly, we measured zeocin cytotoxicity on PNPase mutants by plating Δ*pnp* strains non complemented or complemented with ectopically expressed wild type and mutant *pnp* alleles in the presence of increasing concentration of the drug. As shown in Fig. 1, the Δ*pnp* mutant was slightly more sensitive than the isogenic *pnp* ^+^ strain. Complementation by the wild type allele expressed from plasmid pAZ101, increased the resistance to zeocin to a higher level than the wild type parental strain (Fig. 1a), possibly due to a copy-number effect.

We also tested whether Δ*pnp* mutant complementation for zeocin sensitivity required PNPase catalytic and/or RNA binding activities. As shown in Fig. 1a, i) the zeocin survival curves of the two strains expressing *pnp* mutants lacking a single RBD were similar to that of the wild type-complemented strain; ii) zeocin survival of the strain ectopically complemented with a *pnp* allele lacking both KH and S1 domains was intermediate between the wild type and the Δ*pnp* mutant strains complemented by wild type and single RBD mutant-PNPase; iii) remarkably, the zeocin survival curve of the strain expressing the enzymatically inactive allele *pnp* ^S438A^ was superimposable to that of the non-complemented Δ*pnp* mutant. These results suggest that PNPase enzymatic activity is absolutely required to complement zeocin sensitivity, whereas the RBDs appear to modulate and/or participate to some extent in the overall response to the drug.

RNase II overexpression is known to suppress some of the traits associated to the pleiotropic PNPase deficiency [2, 30]. As shown in Fig. 1b, ectopic expression of RNase II increased zeocin resistance of the Δ*pnp* mutant more than PNPase complementation, thus indicating that RNase II can compensate for PNPase deficiency. Somewhat surprisingly, a non-complemented Δ*rnb* mutant was more resistant than the wild type strain. This could be explained by the fact that PNPase expression level is higher in RNase II-deficient mutants [31] and fits with the observation that ectopic overexpression of PNPase did not substantially alter the survival curve of the Δ*rnb* strain.

### Frequency of mutants induced by zeocin is decreased in *E. coli* Δ*pnp*

In addition to DSBs, the radiomimetic zeocin may cause other types of damage [29] and the survival rate may be the results of different processes in which PNPase may directly or indirectly be implicated. Ideally, a DNA repair process may either restore the original genetic information or introduce mutations [18, 32–35] and inactivation of error free and error prone pathways may lead to increase and decrease of mutation rate, respectively. To specifically analyze whether PNPase participates in repairing DNA DSBs, we assessed the frequency of mutants induced by zeocin treatment. Exponentially growing cultures treated with 100 μg/ml zeocin for 30 min and the non-treated controls were plated on LD-agar plates without and with 100 μg/ml rifampicin to assay for viable cells and rifampicin resistant (Rif^R^) mutants, respectively, as described in Materials and Methods. In these experiments survival of zeocin treated cells was from 30 to 40%.

The ratio of zeocin-induced mutants to the spontaneous mutants (fold induction) in the different strain and the ratio of the fold induction of each strain relative to the control (Δ*pnp* mutant ectopically complemented by the wild type allele) are reported in Table 3. A four-fold induction of Rif^R^ mutant was observed in both the control (C-5691/pAZ101) and wild type (C-1a/pGZ119HE9 strains upon zeocin treatment, whereas this value was reduced to less than a half in the Δ*pnp* strain non-complemented or complemented with a PNPase defective in the catalytic activity. Rif^R^ mutants induction was either comparable to or even higher than the control in Δ*pnp* strains complemented by PNPase lacking both or either KH and S1 RBDs. On the contrary, RNase II did not seem to affect zeocin-induced mutagenesis as a Δ*rnb* mutant, like the control strain, exhibited a fourfold increase of zeocin-induced Rif^R^ mutants. Likewise, ectopic expression of RNase II did not suppress the phenotype of the Δ*pnp* strain.

**Table 3:** Zeocin-induced mutagenesis in Δ*pnp* strains

Strain	Chrom. alleles	Plasmid allele	Fraction surviving (±SD)	Spontaneous mutants (×10^9^)	Zeocin-induced mutants (×10^9^)	Fold induction	Ratio to *pnp* ^+^
C-1a/pGZ119HE	wild	none	0.40 (±0.014)	273.7 (±44.6)	1116.3 (±125)	4.08	0.95
C-5691/pAZ101	Δ*pnp*	*pnp* ^+^	0.37 (±0.028)	77.4 (±19.6)	331.4 (±82.8)	4.28	1.00
C-5691/pGZ119HE	Δ*pnp*	none	0.38 (±0.015)	278.2 (±70.8)	530.7 (±98.0)	1.91	0.45
C-5691/pAZ1112	Δ*pnp*	*pnp*S438A	0.40 (±0.026)	123.9 (±55.2)	222.4 (±85.2)	1.80	0.42
C-5691/pAZ1113	Δ*pnp*	*pnp*-ΔKH	0.31 (±0.073)	15.8 (±11.7)	108.0 (±75.9)	6.81	1.59
C-5691/pAZ1114	Δ*pnp*	*pnp*-ΔS1	0.39 (±0.035)	16.7 (±7.8)	119.0 (±55.4)	7.14	1.67
C-5691/pAZ133	Δ*pnp*	*pnp*-ΔKHS1	0.33 (±0.035)	49.6 (±14.2)	208.4 (77.3)	4.20	0.98
C-5691/pAZ1115	Δ*pnp*	*rnb* ^+^	0.41 (±0.023)	97.4 (±15.9)	189.3 (±16.2)	1.94	0.45
C-5981/pAZ1115	Δ*rnb*	*rnb* ^+^	0.39 (±0.029)	48.6 (±23.8)	204.0 (±85.1)	4.20	0.98
C-5981/pGZ119HE	Δ*rnb*	none	0.36 (±0.027)	228.7 (±50.8)	992.5 (±222.8)	4.34	1.01
C-5981/pAZ101	Δ*rnb*	*pnp* ^+^	0.38 (±0.019)	153.9 (±38.1)	638.4 (±159.4)	4.15	0.97

Overall these data suggest that PNPase participates with its catalytic activity in some zeocin-induced error prone repair pathways, whereas the RBDs are not required for such an activity. On the contrary, RNase II does not seem to differentially affect zeocin-induced error prone and error free DNA repair processes.

### Catalytic and binding activities of KH and S1 mutant PNPase with RNA and DNA substrates

PNPase could indirectly participate in repair of zeocin-induced damage by regulating the expression of genes mechanistically implicated in this process at the level of mRNA stability. Alternatively, PNPase could directly be involved in DNA repair mechanisms. It was shown that the RBDs of PNPase from *Mycobacterium smegmatis* (*Ms*PNPase) differently affect the catalytic and binding activities of the enzyme on RNA or DNA substrates [15, 36]. We thus explored whether the biochemical properties of the KH-S1 truncated PNPase from *E. coli* (*Ec*PNPase) on the two substrates could provide support to either of the above hypotheses. We compared the ability of wild type and RBDs mutant PNPase to degrade RNA and DNA oligonucleotides and to use them as primers for template-independent ribo- and deoxyribonucleotide polymerization. RNA and DNA oligonucleotide-primed reactions were performed in the presence of Mg and Mn divalent cations, respectively.

**Fig. 2:**
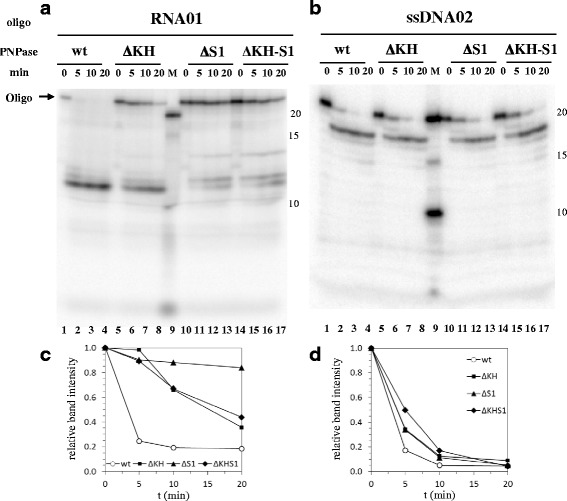
Phosphorolysis of RNA and ssDNA oligonucleotides by PNPase mutants in the RNA binding domains. [^32^P]-5'-end labeled RNA (RNA01; panel **a**) or ssDNA (DNA02; panel **b**) 20-mers, 4 nM each, were incubated at 26 °C with purified His-tagged PNPase or PNPase mutants in the RBDs (10 nM each) in buffer **a** with 10 μM Pi for the times indicated. The reaction products were fractionated by denaturing 6% PAGE. The oligonucleotide band intensities were evaluated by ImageQuant, normalized to the intensity of the 0 min sample, and plotted versus the time. Data from panels **a** and **b** are shown in panels **c** and **d**, respectively

**Fig. 3:**
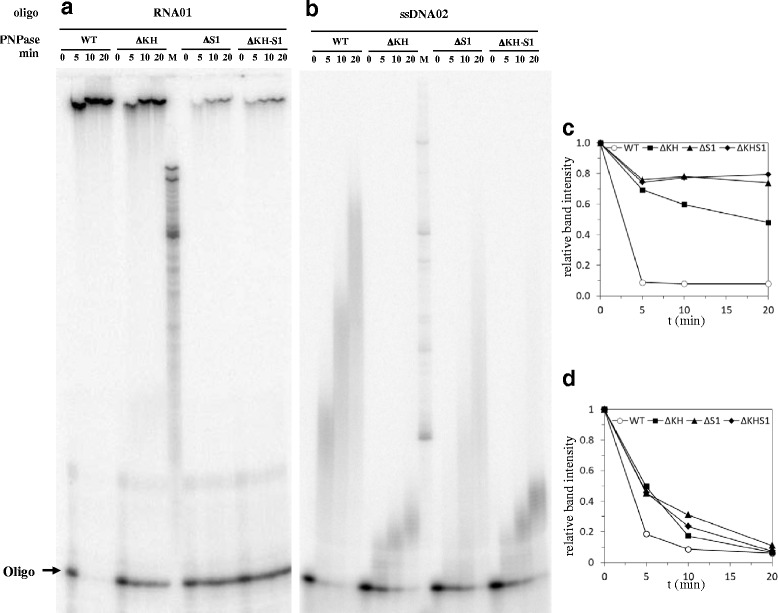
Template-independent synthesis of ssDNA or RNA catalyzed by different purified His-tagged PNPases. [^32^P]-5'-end labeled RNA (RNA01; panel **a**) or ssDNA (DNA02; panel **b**) 20-mers, 4 nM each, were incubated at 26 °C with purified His-tagged PNPase or PNPase mutants in the RBDs (10 nM each) in buffer **a** with 100 μM ADP (Panel **a**) or 100 μM dADP (for ssDNA Panel **b**) for the times indicated. The reaction products were fractionated by denaturing 6% PAGE. The oligonucleotide band intensities were evaluated by ImageQuant, normalized to the intensity of the 0 min sample, and plotted versus the time. Data from panels **a** and **b** are shown in panels **c** and **d**, respectively

As shown in Fig. 2a, the lack of either or both RBDs severely impaired degradation of the ribo-oligonucleotide, whereas degradation efficiency of the DNA oligonucleotide appeared to be only slightly reduced (Fig. 2b; compare the disappearance of the full length probe intensities in mutant vs wild type enzyme lanes, quantified in Fig. 2c and d). Figure 3a shows that deletion of either or both RBDs severely impaired the initiation efficiency of template-independent polymerization of ribonucleotides using an RNA primer and ribonucleotide diphosphates as substrates (see the rate of disappearance of the RNA primer in Fig. 3c) without seemingly affecting the reaction processivity, as indicated by the high molecular weight of the reaction products. On the contrary, using a DNA primer and deoxyribonucleotide diphosphates as substrates, the lack of the RBDs only slightly reduced the efficiency of polymerization initiation whereas it strongly impaired processivity/elongation rate, as evaluated by the rate of disappearance of the DNA oligonucleotide signal and by the average length of the reaction products, respectively (Fig. 3b and d). Noticeably, although deletion of either RBDs impaired to some extent DNA polymerization initiation efficiency (as indicated by the rate of disappearance of the primer), the KH domain appeared to play the major role in processivity/elongation.

**Fig. 4:**
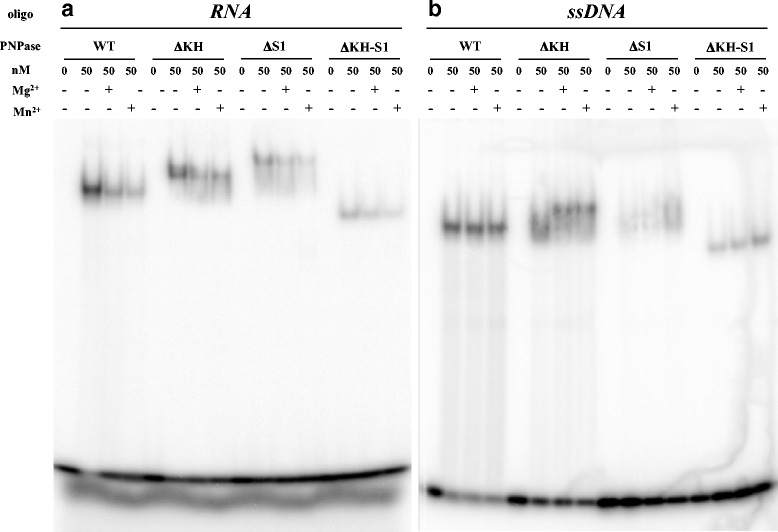
Electrophoretic mobility shift assay of PNPase with RNA and DNA oligonucleotides. [^32^P]-5'-end labeled RNA (RNA01; panel ﻿**a﻿**) or ssDNA (DNA02; panel **﻿b**﻿) 20-mers were incubated 20 min at 21 °C with wild type or ΔKHS1 mutant His-tagged PNPase at the concentrations indicated on the *top* of the panels. 2 mM MnCl_2_ or MgCl_2_ were added to Binding Buffer as specified. PNPase-nucleic acid complexes were resolved by native 5% PAGE. After electrophoresis the gels were dried and the autoradiographic images analyzed by phosphorimaging and ImageQuant software

As for the RNA and DNA binding activities of the PNPase mutants in the RBDs, we performed electrophoretic mobility shift assays (EMSA) on RNA and DNA in the presence of Mg^2+^ and Mn^2+^, respectively (Fig. 4). In the absence of both divalent cations, deletion of the S1 or both RBDs impaired RNA binding, whereas a milder effect was observed with the ΔKH mutant. Surprisingly, each divalent cation reduced binding to the RNA oligonucleotide of both wild type and mutant PNPases (Fig. 4a). In agreement with previous observation [37], wild type PNPase also bound with similar efficiency to the DNA oligonucleotide. However, divalent cations did not reduce PNPase binding to the DNA probe. The lack of the KH domain only mildly affected DNA binding that, on the contrary, was impaired by deletion of S1 or both RBDs. Noticeably, Mn^2+^ seemed to improve DNA binding to the ΔS1 and ΔKH-S1 mutant enzymes (Fig. 4b). Overall these data suggest that S1 domain contributes to ssDNA binding and to processivity of DNA synthesis without a marked effect on either polymerization or phosphorolysis efficiency; on the other end, as previously shown, lack of RBDs not only impairs RNA binding but also PNPase catalytic activity (see [2]).

## Discussion

In this work we present evidence that different features of PNPase participate in HR upon P1 transduction and in error prone DNA repair of DSBs induced by zeocin in *E. coli*. Although PNPase is not essential for these processes, its contribution was readily and consistently detected. PNPase is implicated in mRNA and sRNA decay and processing, thus controlling the expression of several genes by various mechanisms [2]. A key question is whether PNPase may directly participate in recombination and repair pathways or indirectly controls these processes by regulating the expression of other genes directly involved in recombination and repair, and/or through metabolic pathways. This issue will be discussed below separately for recombination and repair.

### PNPase structurally participates in HR protein complexes and modulates HR via its KH and S1 domains

We show that in *E. coli* mutants lacking PNPase P1 phage-mediated transduction frequency is reduced by up to five-six folds. Transductional recombination is thought to mainly depend on RecBCD recombination pathway, whereas the RecF pathway seems to be responsible for less than 10% of the recombinants [26]. However, only a minor fraction of the transduced DNA undergoes recombination and gives rise to recombinant progeny. On the contrary, up to 90% of the total remains within the cytoplasm in a stable, supercoiled form, likely linked to specific phage encoded protein (s), and neither replicates nor is degraded for at least 5 h after infection (abortive transduction). Full transduction frequency may be increased at the expenses of abortive transduction by UV irradiating the donor strain or the phage lysate and by phage mutations [26]. We cannot rule out that PNPase regulates transduction efficiency by converting the abortive form of transduced DNA into the recombination proficient configuration. We favour, however, a hypothesis that implicates PNPase in the recombination pathway. In mutants expressing a PNPase devoid of catalytic activity but conserving the KH and S1 RBDs [24] transduction frequency was only marginally affected (Table 2). This suggests that PNPase is not implicated in transduction via processing of recombination intermediates or degradation of specific mRNAs coding for proteins implicated in this process. On the other end, transduction frequency was reduced 3–5-folds in mutants in either RBD, thus suggesting that PNPase may play a regulatory role, possibly through interactions with RNA or DNA. Surprisingly, deletion of both KH and S1 domains dramatically impaired transduction frequency much more than the lack of PNPase itself. A working model to rationalize such results is that PNPase associates with other elements of the HR machinery through its structural core and facilitates some steps of the recombination process through its RBDs. The lack of both domains may not impair the association of the PNPase core with the component (s) of the recombination machinery but would poison the complex, thus inhibiting recombination.

Interestingly, it was observed that *E. coli* RecA protein filaments may contain RNA and a putative PNPase activity [38, 39]. These observations lend support to the idea that in *E. coli* PNPase may interact with elements of the recombination machinery and regulate their function independently of its catalytic activity. In contrast with this hypothesis Rath et al. [16], based on the observation that the recovery of recombinants was 1–2% of the recipient number for both wild and *pnp* mutant strains, claim that conjugational recombination is unaffected by PNPase. Unfortunately these authors did not provide sufficient details, such as the donor to recipient ratio, to critically evaluate their results. Anyway, it should be noted that conjugation and generalized transduction, although largely dependent on the RecBCD pathway, differ in several features that might affect some critical steps. For example, whereas in transduction a linear dsDNA molecule is injected by P1, upon conjugation DNA is processively transferred into the recipient as a single stranded molecule with its 5′-end covalently linked to the relaxase to be then replicated discontinuously [40]. Therefore a different involvement of PNPase in transduction- and conjugation-driven HR could be justified.

### PNPase participates in error-prone DSBR with its catalytic activity

Recombination and DNA repair are intimately intertwined processes. In *E. coli* and several other bacteria one of the most relevant recombination pathways is the RecBCD/AddAB/AdnAB-dependent DSBR system, which can fix DNA breakages in an error free manner [41]. On the other side, template-independent non-homologous end joining (NHEJ) pathways can repair chromosome breaks at the cost of mutations [42]. Although evolutionarily conserved, the classical NHEJ pathway is missing in many bacteria such as *E. coli*, which lacks the gene encoding two signature factors of the NHEJ system, namely the Ku and ligase D proteins [42]. Nevertheless, an alternative template-independent pathway, termed alternative-end joining (A-EJ) appears to be operating in *E. coli* [43].

To test whether and how PNPase could participate not only in HR but also in DNA repair we assessed in different PNPase mutants the frequency of Rif^R^ mutants induced by zeocin, a radiomimetic that causes DNA DSBs in vivo [29]. In our experimental conditions we observed that induction of Rif^R^ mutants in strains lacking PNPase or expressing a catalytically inactive enzyme is about the half than in strains expressing the wild type protein or variants lacking either or both RBDs (Table 3). It thus appears that PNPase is implicated in a mutagenic DSB repair pathway and that, contrary to what we observed in HR, its catalytic activity is strictly required. Obviously these data do not rule out that PNPase may be also implicated in error free repair pathways through HR and that the overall effect could be the summation of both (or more) processes.

It should be noted that deletion of PNPase RBDs strongly impairs RNA degradation and template-independent polymerization efficiency, whereas it affects to a lesser extent these catalytic activities on DNA substrates (only polymerization processivity appears to be strongly impaired; Figs. 2 and 3). Since PNPase catalytic activity is required for induction of mutants, these observations fit the hypothesis that PNPase is directly implicated in DNA transactions in an error prone repair pathway rather than controlling expression of a DNA repair pathway at the level of mRNA stability. In addition, the lack of PNPase RBDs (in particular the KH domain) impairs in vitro polymerization (in particular polymerization processivity) with dNTPs as a substrate, whereas it seems to minimally affect ssDNA phosphorolysis (Figs. 2 and 3). It might thus be inferred that ssDNA degradation rather than synthesis is required for the mutagenic pathway. For example, PNPase might participate, together with RecBCD, in resection of single strand protruding ends and/or could remove “dirty” (not ligatable) ends thus contributing to generate substrates for the A-EJ pathway. We cannot rule out, however, that the residual template-independent polymerizing activity of PNPase lacking the RBDs may suffice to support the mutagenic repair pathway and that both (limited) resection and synthesis of ssDNA ends may be implicated in the process.

In *B. subtilis* PNPase copurifies with RecN, a key protein for the repair of DNA DSBs [44], and it is required for the formation of RecN-promoted discrete repair centers upon DSBs induction. In this context PNPase provides the RecN-associated 3′ → 5′ ssDNA exonucleolytic activity. Moreover, PNPase catalytic activity on ssDNA is modulated in vitro by RecN, RecA and SSB [13, 14]. Thus PNPase from a distantly related bacterium seems to physically and functionally interact with recombination and DSB repair systems.

It has been shown [15, 36] that in *Ms*PNPase deletion of the RBDs impairs RNA phosphorylase and polymerase activities whereas it enhances the DNA polymerase and phosphorylase activity. Moreover, lack of the S1 RBD enhances divalent cation-dependent (catalytic) binding to ssDNA and DNA polymerase activity, thus suggesting that the S1 domain of *Ms*PNPase on the one hand helps capturing an RNA polynucleotide substrate for processive 3′ end polymerization, on the other one provides a specificity filter that selects against a DNA polynucleotide substrate. *Ec*PNPase, however, seems to differ from *Ms*PNPase in that deletion of RBDs seems to impair, albeit mildly, PNPase catalytic activity on a DNA substrate, with a marked reduction of polymerization processivity. In Mycobacteria three distinct pathways are known to participate in DSB repair of DNA, namely HR, NHEJ and single-strand annealing [45]. It is possible that differences in biochemical properties related to DNA transactions of PNPase from these two distantly related bacteria may reflect different roles of this enzyme in the recombination and repair pathways.

Overall our data suggest that in *E. coli* PNPase may participate to recombination and repair pathways in different ways. PNPase may interact with HR pathways and modulate some steps through its RBDs, whereas its catalytic activity does not appear to be implicated in this process. On the other hand, this enzyme may participate in an error-prone DSB repair process through its catalytic activity.

PNPase has been previously implicated also in mutagenesis caused by spontaneous misincorporation errors that occur during replication and are normally corrected by the mismatch repair (MMR) systems. Genetic interactions between *pnp* and the MMR system encoded by *mutS*, *mutL*, *mutH* and *uvrD* have been shown by both Jawali and coworkers [16] and by Miller and coworkers [17]. The former authors found that deletion of *pnp* partially suppresses the mutator phenotype of a *uvrD* deficient mutant and suggested that PNPase may regulate the expression of a hypothetical functional homologue of *uvrD*. The latter group showed that deletion of *pnp* suppresses the mutator phenotype of *mutS* or *mutL* mutants. The underlying mechanism suggested by these authors [17] is that phosphorolysis, by generating ribonucleotides diphosphates that serve as substrates for the salvage biosynthetic pathways of dNTPs, affects the nucleotide pool concentration and the rate of base misincorporation during replication [46, 47]. Thus PNPase might contribute in different ways to different mutagenic pathways.

### Cytotoxicity of genotoxic agents in *pnp* mutants

Genotoxicity of toxic chemicals may account for only a quota of their cytotoxicity [48]; therefore, survival of cells exposed to genotoxic agents may also depend on mechanisms not implicated in DNA repair. We have shown that PNPase features required to complement a Δ*pnp* mutant for zeocin resistance are not completely superimposable to those required for zeocin mutagenesis or HR. Notably, zeocin sensitivity of *pnp* mutants, unlike zeocin-induced mutagenesis, is suppressed by overexpression of RNase II, thus suggesting that, at least in part, zeocin detoxification occurs via post-transcriptional control of gene expression and/or degradation of damaged RNA.

PNPase deficient mutants have been shown to be more sensitive to genotoxic agents in different species. *E. coli* mutants are more sensitive to hydrogen peroxide treatment which increases the levels of oxidized ribo- and deoxyriboguanosines; moreover, *E. coli* PNPase was suggested to contribute to cell survival to oxidative stress by acting as a specific scavenger of oxidized RNA [49]. *E. coli pnp* mutants also exhibit UV sensitivity. This phenotype is not epistatic to the *uvrABC* nucleotide excision repair (NER) system and to *recJ*, *recQ*, *recG*, thus suggesting that PNPase is implicated neither in NER nor in the single-stranded gap repair. However, UV sensitivity of *pnp* mutants is epistatic to *uvrD*, *recB* and *ruvA*, thus implicating PNPase in the recombinational repair process [16]. *B. subtilis* lacking PNPase is more sensitive to oxidative stress (chronic exposure to H_2_O_2_) but shows increased tolerance to other DNA damaging agents such as methyl methane sulfonate, 4-nitroquinoline-1-oxide or mitomycin C, as compared to wild type cells [13]. It thus appears that different repair and detoxification pathways may be differently activated or repressed by PNPase.

## Conclusions

The scenario emerging from our results and those discussed hereby is that in *E. coli* various features of PNPase influence several different pathways implicated in DNA metabolism, such as homologous and non-homologous recombination, DNA repair, spontaneous and induced mutagenesis through a variety of mechanisms. RNA degradation may not only modulate expression of genes implicated in the above processes, but also impact the relative composition of nucleotide pool, thus affecting the rate of misincorporation at replication, and destroy RNA damaged by genotoxic agents, which may be toxic to the cell. In addition, as this enzyme can both degrade and synthesize ssDNA in a template-independent manner, it may directly participate in error-prone repair pathways that depend on these features. Finally, PNPase appears to non-enzymatically interact with components of HR machinery whereby modulating HR efficiency via its nucleic acid binding activity. It is remarkable that this non-essential gene has evolved so many subtle regulatory interactions with core cellular processes implicated in RNA and DNA metabolism.

## Methods

### Bacterial strains and media

Bacterial strains and plasmids are described in Tables 4 and 5, respectively. Recombinant plasmids used in complementation experiments were constructed in pGZ119HE [50], a derivative of the ColD high copy number plasmid (15–20 plasmids per chromosome in the exponential phase [51]). Unless otherwise stated, bacteria were grown at 37 °C in LD broth and LD agar plates [52]; auxotrophy screenings and selections were performed in M9-agar minimal medium [24]. Culture media were supplemented, as needed, with 0.2% arabinose, 0.2% glucose, 100 μg/ml ampicillin, 30 μg/ml chloramphenicol, 50 μg/ml kanamycin, 50 μg/ml streptomycin, 100 μg/ml rifampicin, 0.1 mM IPTG, and zeocin (InvivoGen) at the concentrations indicated.

**Table 4:** Bacterial strains

Strain	Parental	Relevant characters	Construction/Reference
BW25113	Prototype	*E. coli* K12	[54]
C-1a	Prototype	*E. coli* C, prototrophic	[55]
C-5691	C-1a	∆*pnp751*	[56]
C-5883	C-1a	∆*leuA*::*kan*	by P1HFT * JW0073 transduction; this work
C-5884	C-5691	∆*leuA*::*kan* ∆*pnp751*	by P1HFT * JW0073 transduction; this work
C-5885	C-1a	∆*trpE*::*kan*	by P1HFT * JW1256 transduction; this work
C-5886	C-5691	∆*trpE*::*kan* ∆*pnp751*	by P1HFT * JW1256 transduction; this work
C-5981	C-1a	Δ*rnb::kan*	by P1HFT * JW1279 transduction; this work
JW0073	BW25113	∆*leuA*::*kan*	[57]
JW1256	BW25113	∆*trpE*::*kan*	[57]

**Table 5:** Plasmids

Plasmid	Relevant characters	Reference
pAZ101	pGZ119HE derivative, harbours the *pnp* ^+^ allele	[58]
pAZ1112	pAZ101 derivative; harbours the *pnp*-S438A allele encoding a catalytically inactive PNPase	[24]
pAZ1113	pAZ101 derivative; harbours the *pnp*-*74* allele (ΔKH 603–615) under the control of *pnp* P2 promoter. Obtained by cloning the *Age*I-*Bsi*WI fragment of *pnp*-74 from pEJ04 in the large *Age*I-*Bsi*WI fragment of pAZ101.	this work
pAZ1114	pAZ101 derivative; harbours the *pnp*-*78* allele (ΔS1 622–633) under the control of *pnp*-*p2* promoter. Obtained by cloning the *Age*I-*Bsi*WI fragment of *pnp*-78 from pEJ08 in the large *Age*I-*Bsi*WI fragment of pAZ101.	this work
pAZ1115	pGZ119HE derivative; harbours the *rnb* ^+^ allele under the control of its own *rnb-p* promoter. Obtained by cloning into pGZ119HE a *Bam*HI-*Hin*dIII-digested PCR fragment amplified from MG1655 DNA with primers FG3063 (CG**GGATCC**TGCAAGGGCGAAAATG) and FG3086 (CCC**AAGCTT**CATGAAATTAACGGCGGC) encompassing chromosomal coordinates 1,349,209-1,344,800 (NCBI Sequence ID U00096.3).	this work
pAZ133	pAZ101 derivative, harbours the Δ*pnp*-833 allele encoding Pnp-ΔKHS1	[23]
pEJ04	Harbours the *pnp*-74 allele under T5 promoter-lacO operator control	[22]
pEJ08	Harbours the *pnp*-78 allele under T5 promoter-lacO operator control	[22]
pGZ119HE	ColD, Cam^R^	[50]

### Transduction

P1 HFT plate stocks were grown on the prototrophic donor strain C-1a from a single plaque as described by Miller [53]. The auxotrophic recipient strains were grown in LD broth with 5 mM CaCl_2_ at 37 °C up to 0.5 OD_600_, spun down (4500 rpm for 10 min at 4 °C), resuspended in 1/10 volume of MC buffer (10 mM MgSO_4_; 5 mM CaCl_2_), and incubated 30 min at 37 °C with aeration. Cells were infected with P1 HFT at a multiplicity of infection (m.o.i.) of 0.5 at 37 °C. To determine unadsorbed phage titer, 20 min upon infection a 0.02 ml sample was transferred into a tube with 2 ml of ice-cold M9-citrate buffer (10 mM Na-citrate in M9 medium) and a drop of chloroform, vigorously shaken, centrifuged 5 min at 13,000 rpm, and the supernatant assayed for plaque forming units. At the same time the infected cells were diluted 5-fold in M9-citrate buffer, washed twice in one volume and resuspended in 1/10 volume of the same buffer. The prototrophic transductants were then selected by plating on M9-glucose agar plates. Loss of the Kan^R^ marker associated to auxotrophy was assayed by replica plating of either all transductants obtained, if the total number was smaller than 45, or at least 45 individual transductant colonies per each transduction. Frequency of transduction was calculated as the ratio of auxotrophic transductants to the adsorbed phage.

### Zeocin-induced mutagenesis

Bacterial cultures grown in LD at 37 °C with aeration up to OD_600_ = 0.25 were split in two aliquots and zeocin (100 μg/ml as indicated) was added to one culture. After 30 min of further incubation, both cultures were washed twice by centrifugation and resuspended in 400 μl of LD broth. 10 μl were used to assay the total viable counts on LD agar, whereas the remaining was plated on four LD-agar plates supplemented with 50 μg/ml rifampicin. Rif^R^ mutants were scored after 48 h incubation at 37 °C. For strains harbouring a plasmid, the growth media were supplemented with 30 μg/ml of chloramphenicol. Mutants frequency was assessed from three independent cultures.

### EMSA and in vitro RNA degradation and polymerization assays

^32^P-radiolabelled RNA and DNA probes for EMSA and in vitro degradation and polymerization assays were prepared as follows. 10 pmol of a 20-mer RNA oligonucleotide (RNA01: 5′-ACUGGACAAAUACUCCGAGG-3′ [24]; and a 20-mer ssDNA oligonucleotide (DNA02: 5′-ACTGGACAAATACTCCGACG-3′; were labelled at their 5′ end with [γ-^32^P] ATP by T4 polynucleotide kinase (New England Biolabs) in 20 μl of polynucleotide kinase buffer provided by the manufacturer; after ethanol precipitation in the presence of 1 mg/ml glycogen to remove non-incorporated nucleotides, the samples were suspended in the same volume of RNase-free water. For EMSA, 50 fmol of either RNA01 or DNA02 probes were incubated for 20 min at 21 °C in Binding Buffer (50 mM Tris HCl pH 7.4, 50 mM NaCl, 0.5 mM DTT, 0.025% NP40 (Fluka), 10% glycerol) with increasing amounts of purified His-tagged PNPases in a final volume of 10 μl. The samples were fractionated by native 5% polyacrylamide gel electrophoresis (PAGE) at 4 °C. For in vitro template independent polymerization experiments, 80 fmol of radiolabelled RNA or ssDNA primers were incubated in buffer A (10 mM Tris–HCl, pH 7.5, 10 mM KCl, 2 mM MgCl_2_ (for RNA) or 2 mM MnCl_2_ (for ssDNA), 0.75 mM DTT, 2% PEG-6000) containing 100 μM ADP (for RNA) or 100 μM dADP (for ssDNA) and 10 nM PNPase at 26 °C in a final volume of 20 μl. For in vitro degradation experiments, 80 fmol of radiolabelled RNA or ssDNA primers were incubated in buffer A with 10 μM Pi and 10 nM PNPase at 26 °C in a final volume of 20 μl. Three μl aliquots were withdrawn at different time points, diluted in 5 μl of RNA loading dye (2 mg/ml xylene cyanol and bromophenol blue, 10 mM EDTA in formamide) and fractionated by denaturing 6% PAGE. After electrophoresis the gels were dried and the autoradiography images analyzed by phosphorimaging and ImageQuant software.

## Abbreviations

A-EJ: Alternative-end joining
CFU: Colony forming unit
dNDP: Deoxyribonucleotide
dNTP: Deoxyribonucleoside triphosphate
DSB: Double strand break
DSBR: Double strand break repair
dsDNA: Double strand DNA
DTT: Dithiothreitol
e.o.p.: Efficiency of plating
*Ec*PNPase: PNPase from *E. coli*
EMSA: Electrophoretic mobility shift assays
HR: Homologous recombination
IC_1_: Inhibitory concentration giving 1% survival
IPTG: Isopropyl β-D-1-thiogalactopyranoside
Kan^S/R^: Kanamycin sensitive/resistant
m.o.i.: Multiplicity of infection
MMR: Mismatch repair
*Ms*PNPase: PNPase from *M. smegmatis*
NDP: Nucleoside diphosphate
NER: Nucleotide excision repair
NHEJ: Non-homologous end joining
OD_600_: Optical density at 600 nm wavelength
PAGE: Polyacrylamide gel electrophoresis
PAP: Polyadenylpolymerase
Pi: Inorganic phosphate
PNPase: Polynucleotide phosphorylase
RBD: RNA binding domain
Rif^R^: Rifampicin resistant
rpm: Revolutions min^−1^
sRNA: Small RNA
ssDNA: Single strand DNA
TF: Transduction frequency
Tris: Tris (hydroxymethyl) aminomethane
UV: Ultraviolet light
Δ: Deletion
